# Influenza vaccine and subsequent development of zoster

**DOI:** 10.1111/irv.13055

**Published:** 2022-09-17

**Authors:** Kai‐Che Wei, Yu‐Chia Chang, Yu‐Tung Huang

**Affiliations:** ^1^ Department of Dermatology Kaohsiung Veterans General Hospital Kaohsiung Taiwan; ^2^ School of Medicine National Yang Ming Chiao Tung University Taipei Taiwan; ^3^ Department of Long‐Term Care, College of Health and Nursing National Quemoy University Kinmen County Taiwan; ^4^ Department of Healthcare Administration, College of Medical and Health Science Asia University Taichung Taiwan; ^5^ Center for Big Data Analytics and Statistics, Department of Medical Research and Development Chang Gung Memorial Hospital Linkou Main Branch Taoyuan Taiwan; ^6^ Department of Health Care Management Chang Gung University Taoyuan Taiwan

**Keywords:** herpes zoster, influenza vaccine, side effect

## Abstract

**Background:**

Herpes zoster (HZ), which is caused by reactivation of the latent varicella zoster virus, was not listed as a side effect of any vaccines until the introduction of coronavirus disease 2019 (COVID‐19) vaccine. This study used a nationwide population database to examine whether the HZ risk is increased after receiving the influenza vaccination.

**Methods:**

This population‐based retrospective self‐controlled case series evaluated the association between influenza vaccine exposure and HZ risk. Data were collected from Taiwan's National Health Insurance Research Database between 2015 and 2017. Patients with HZ diagnosed within 6 months before and after receiving the influenza vaccination were included. After receiving the influenza vaccine, the first 15 and 30 days were defined as risk intervals, while the other periods were defined as control intervals. Poisson regression was used to compare the incidence rate ratio (IRR) for HZ during the risk interval vs. the control interval.

**Results:**

In total, 13,728 patients were diagnosed with HZ before and after receiving the influenza vaccine. The IRR for days 1–15 was significantly higher (IRR = 1.11; 95% confidence interval [CI], 1.02–1.20), but insignificant for days 1–30 (IRR = 1.04; 95% CI, 0.98–1.10). In a subgroup analysis, the IRRs were significantly higher in participants, including 50–64 years old (1.16; 95% CI, 1.02–1.33), males (1.14; 95% CI, 1.01–1.28), and healthier individuals (i.e., no history of cancer or autoimmune diseases).

**Conclusions:**

There was a slight increase in risk of HZ in people receiving influenza vaccine in the first 1–15 days after vaccination.

## 1 INTRODUCTION

Herpes zoster (HZ), also commonly known as shingles, is caused by reactivation of the latent varicella zoster virus (VZV), and occurs more commonly in the elderly and immunocompromised people.^1^ HZ is not a deadly disease; however, its complications, such as herpes zoster ophthalmicus and postherpetic neuralgia (PHN), can exert a serious impact on the health and quality of life of patients with HZ.^2^ Moreover, HZ usually causes unnecessary panic among patients.

HZ was not listed as a side effect of any vaccines (except for occasional case reports) before the coronavirus disease 2019 (COVID‐19) pandemic.^3^ There are, however, many reports of increased risk for HZ in people who received the COVID‐19 vaccine,^4^, ^5^, ^6^ especially mRNA‐based vaccines. The exact mechanism whereby vaccines arouse VZV latency remains unclear. Some researchers believe that mRNA vaccines induce immune reconstitution, which has been reported in other types of vaccines, including adenovirus‐ or subunit‐based vaccines.^7^ The phenomenon described for COVID‐19‐associated HZ might be the same for conventional vaccines, e.g., influenza vaccines.

In this study, we used a nationwide population database to explore whether HZ risk increases after receiving the influenza vaccine.

## 2 MATERIALS AND METHODS

### 2.1 Background information

In Taiwan, a single‐payer mandatory enrollment National Health Insurance (NHI) program was launched in 1995, which covered more than 99% of citizens of Taiwan.^8^ The policy of free influenza vaccination for all people aged 65 years and older was implemented in 2001. Other vaccination targets were gradually added, including medical staff, epidemic prevention workers, young children, patients with catastrophic illness, pregnant women, etc. In 2016, in order to protect the health of adults aged 50–64 years, this group was further included in the target of public‐funded vaccines.

The vaccine used in Taiwan is composed of viral strains updated according to the World Health Organization's annual recommendations for the Northern Hemisphere, and its protective efficacy is the same as that of other countries. The publicly funded influenza vaccine used in Taiwan is a trivalent vaccine containing three inactivated viruses: A H1N1, A H3N2, and influenza B. The viral strains for the 2016–2017 Northern Hemisphere influenza vaccine were an A/California/7/2009 (H1N1)‐like virus, A/Hong Kong/4801/2014 (H3N2)‐like virus, and B/Brisbane/60/2008‐like virus (Victoria lineage) in the trivalent formulation (reference: https://www.cdc.gov/flu/about/season/flu-season-2016-2017.htm).

### 2.2 Data source

This population‐based study was a secondary data analysis using nationwide data from Taiwan's NHI research database (NHIRD). The data were released by the Health and Welfare Data Science Center, Ministry of Health and Welfare of Taiwan (HWDC, MOHW). The NHIRD of this study was covered during the 2015–2017 period and comprised the detailed information of beneficiaries enrolled in the NHI program, including clinical records on outpatient visits, hospitalizations, and prescriptions. According to the policies of Taiwan's NHI, medical claims are sent to the Bureau of NHI for cross‐checking and validation to ensure the adequacy of diagnosis coding. Hospitals or clinicals found to have fraudulent coding, overcharging, or malpractice will be penalized or restrained of the treatment fees. Several validation studies have been performed to support the validity of diagnosis codes in the NHIRD.^8^ Owing to the anonymity of the database, the requirement for informed consent was waived. This study was approved following an ethical review conducted by the Institutional Review Board of the Taichung Jen‐Ai Hospital, Taiwan (Institutional Review Board No. 108‐83).

### 2.3 Study design and populations

**FIGURE 1:**
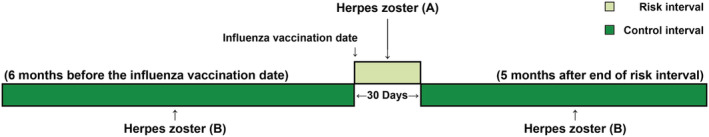
Schematic of study design. “Herpes zoster (A)” represented the elderly who is diagnosed with herpes zoster at any time during the 15‐day and 30‐day risk interval (light‐shaded areas) after influenza vaccination. “Herpes zoster (B)” represented the elderly who is diagnosed with herpes zoster during the control interval (dark‐shaded areas). The study assessed the relative incidence of herpes zoster during the risk interval as compared to that during the control interval.

We used a self‐controlled case‐series design to evaluate the association between influenza vaccination and HZ. The study population comprised patients who had been diagnosed with HZ within 6 months before and after receiving the influenza vaccine in 2016. After excluding patients (1) receiving influenza vaccine more than once (atypical way for influenza vaccine), (2) died within 6 months after receiving influenza vaccine, and (3) with missing value of confounding factors (age, sex, premium‐based monthly salary, and urbanization level), we identified 13,728 patients who were enrolled as study subjects. A schematic algorithm detailing the study selection procedures is shown in Figure 1. Theoretically, if the HZ was not related to influenza vaccination, the incidence of HZ for our selection subjects would presumably be distributed equally across the entire observation period. The first 15 and 30 days after receiving the influenza vaccine were defined as risk intervals, and the other periods (6 months before and 5 months after receiving influenza vaccine) were defined as control intervals.

### 2.4 Definition

To increase the accuracy of diagnosis, HZ was defined as having a primary diagnosis (*International Classification of Diseases, Ninth Revision, Clinical Modification* [ICD‐9‐CM]: 053.x, and *Tenth Revision* [ICD‐10‐CM]: B02.*) and concurrent use of related medications, including acyclovir (anatomic therapeutic chemical [ATC] code: J05AB01), famciclovir (ATC code: J05AB11), and valacyclovir (ATC code: J05AB09). The confounding factors adjusted for in this study included sex, age, premium‐based monthly salary, urbanization level, the Charlson comorbidity index (CCI) score,^11^ comorbid autoimmune diseases, and cancer. CCI scores were calculated using the diagnosis of outpatient or inpatient admission during the preceding 12 months prior to the vaccination date. In addition, we used the registry for catastrophic illness in the NHIRD to define whether subjects had comorbid autoimmune diseases or cancer.

### 2.5 Statistical analysis

We used the Poisson regression to compute the incidence rate ratio (IRR) and 95% confidence interval (CI) for incident HZ during the risk interval compared to that in the control interval. The incidence of HZ per study subject during the observation period was accounted for in the model. We evaluated the risks of incident HZ during days 1–15, and 1–30 after receiving influenza vaccine, respectively. To test the robustness of our findings, we conducted two sensitivity analyses that limited the control interval to the post‐exposure observation time (i.e., 5 months after receiving influenza vaccine) and limited the pre‐exposure observation time to 6 months before receiving influenza vaccine. We also performed a stratified analysis in subgroups defined according to age (<50, 50–64, and ≥65 years), sex, CCI (score 0, 1, and ≥2), autoimmune disease, and cancer (present or absent). Statistical analysis was performed using the sas software Version 9.4 (SAS Institute, Cary, NC, USA). A *p*‐value <0.05 was considered statistically significant.

## 3 RESULTS

Table 1 provides the detailed characteristics of the study participants. In 2016, a total of 3,370,307 individuals in the NHIRD received the publicly funded influenza vaccine, of whom 1,294,590 (38.41) were older than 65 years, 1,080,886 (32.07%) were 50–64 years old, and 29.52% were <50 years old. In total, 13,728 patients presented with incident HZ. The average age across all patients was 65.47 ± 13.43 years. The percentages of participants aged <50, 50–64, and ≥65 years old were 9.38, 33.83, and 56.80%, respectively. Most of the participants (56.37%) were females. In total, 49.2% of the participants had a CCI score ≥2. In addition, 12.19% of the patients had cancer, and 2.43% had a comorbid autoimmune disease.

**TABLE 1:** Baseline characteristics of people receiving influenza vaccination and with incident herpes zoster during the observation period

Variables	Influenza vaccination		Herpes zoster	
	N	%	N	%
Total	3,370,307	100.00	13,728	100.00
Age				
<50	994,831	29.52	1287	9.38
50–64	1,080,886	32.07	4644	33.83
≥65	1,294,590	38.41	7797	56.80
Mean ± SD	54.01 ± 23.32		65.47 ± 13.43	
Sex				
Male	1,507,325	44.72	5989	43.63
Female	1,862,982	55.28	7739	56.37
Premium‐based monthly salary (NTD)				
≤21,000	854,206	25.35	3730	27.17
21,001–22,800	1,006,010	29.85	4763	34.70
22,801–40,100	689,611	20.46	2284	16.64
≥40,101	820,480	24.34	2951	21.50
Urbanization level				
Level 1	862,539	25.59	3467	25.25
Level 2	1,052,328	31.22	3961	28.85
Level 3	572,156	16.98	2140	15.59
Level 4	505,085	14.99	2195	15.99
Level 5	79,338	2.35	389	2.83
Level 6	153,429	4.55	842	6.13
Level 7	145,432	4.32	734	5.35
CCI score				
0	1,450,393	43.03	3726	27.14
1	795,492	23.60	3165	23.06
≥2	1,124,422	33.36	6837	49.80
Cancer				
No	3,172,653	94.14	12,054	87.81
Yes	197,654	5.86	1674	12.19
Autoimmune disease				
No	3,339,999	99.10	13,395	97.57
Yes	30,308	0.90	333	2.43

Abbreviations: CCI, Charlson comorbidity index; NTD, New Taiwan dollar.

The incidence rates (IR) (per 1000 person‐days) of HZ of risk periods for days 1–15 and 1–30 were 3.15 and 2.96. As compared to control periods, the IRs of HZ for post‐exposure observation time and pre‐exposure observation time were 2.88 and 2.81. The IRRs for HZ after influenza vaccination are shown in Table 2. When compared to the control interval, the IRR for days 1–15 (risk interval) was significantly higher (IRR = 1.11; 95% CI, 1.02–1.20) than for days 1–30 (IRR = 1.04; 95% CI, 0.98–1.10). The same results were observed in sensitivity analyses limiting the control interval to the post‐exposure and pre‐exposure observation times.

**TABLE 2:** The incidence rate ratio of incident herpes zoster after influenza vaccination

	Events		Observed person‐days		IRR (95% CI)	P value
	Case	Control	Case	Control		
Primary analysis						
Days 1–15	649	12,884	205,920	4,530,240	1.11 (1.02–1.20)	0.011
Days 1–30	1217	12,884	411,840	4,530,240	1.04 (0.98–1.10)	0.202
Sensitivity analyses						
Control interval limited to post‐exposure observation time						
Days 1–15	649	5935	205,920	2,059,200	1.09 (1.01–1.19)	0.030
Days 1–30	1217	5935	411,840	2,059,200	1.03 (0.96–1.09)	0.418
Control interval limited to pre‐exposure observation time						
Days 1–15	649	6952	205,920	2,471,040	1.12 (1.03–1.21)	0.006
Days 1–30	1217	6952	411,840	2,471,040	1.05 (0.99–1.12)	0.114

Abbreviations: CI, confidence interval; IRR, incidence rate ratio.

**FIGURE 2:**
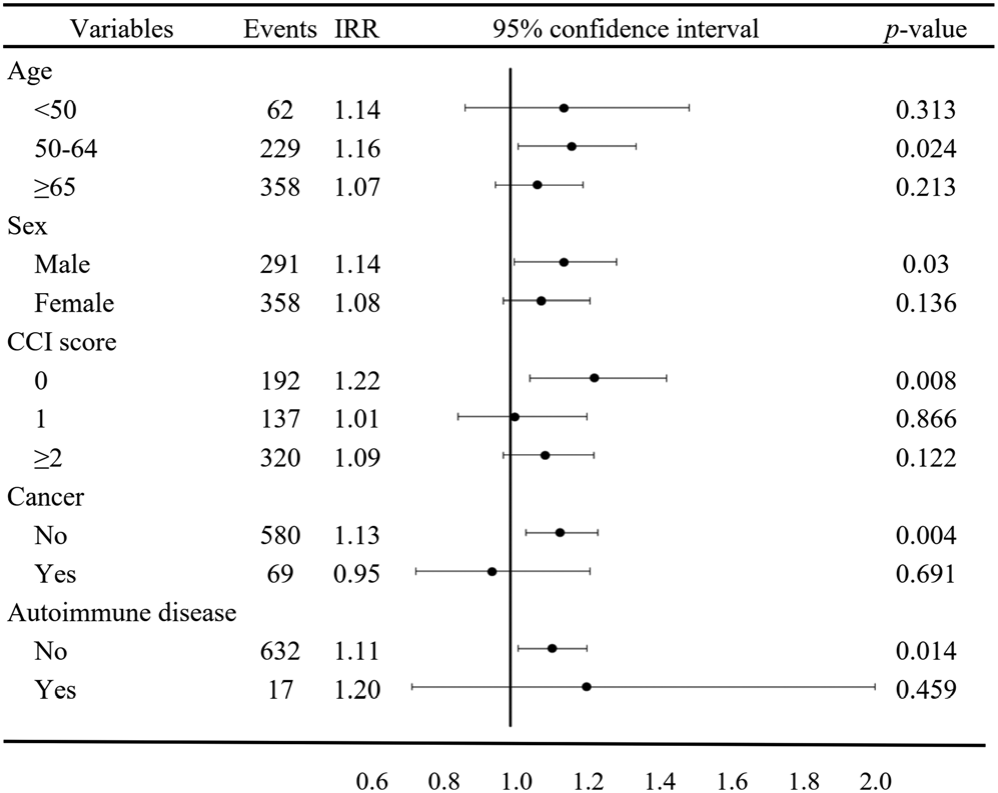
Subgroup analyses comparing incidence rate ratios (IRRs) for incident herpes zoster during days 1–15.

The IRR for HZ after influenza vaccination was significantly higher for days 1–15 compared to that with the control interval, but insignificantly for days 1–30; therefore, we only included days 1–15 for stratified analyses. Figure 2 showed the results of subgroup analyses for the risks of incident HZ during days 1–15. The IRR for 50‐64 years old participants was 1.16 (95% CI, 1.02–1.33) and for males was 1.14 (95% CI, 1.01–1.28). A CCI score of 0 (IRR = 1.22; 95% CI, 1.05–1.14), as well as the absence of comorbid cancer (IRR = 1.133; 95% CI, 1.04–1.23), and autoimmune disease (IRR = 1.11; 95% CI, 1.02–1.20) were associated with an increased risk of developing HZ after receiving the influenza vaccine.

## 4 DISCUSSION

By using a nationwide database, the current study revealed a marginal increase in HZ incidence within 1–15 days after receiving the influenza vaccine in the general population, particularly among individuals aged 50–64 years, as well as in those with few comorbidities, such as cancer and autoimmune diseases. This discrepancy in risk across different age groups and health status is novel and might be due to an increased risk for HZ in older people, which renders the effect of vaccination on HZ risk less apparent. In contrast, the incidence of HZ was significantly low in young people and those that were generally healthy. An increase in the number of HZ cases is therefore relatively easy to observe. Furthermore, although the IRR in patients with autoimmune diseases is not statistically significant, it might be due to the small number of cases contributing to its non‐significance.

Previous studies have identified various factors which can lead to VZV reactivation, including aging, physical or emotional stress, immunosuppression, and some medication, such as Janus kinase inhibitors.^9^, ^10^ It is unlikely that the possible link between HZ and recent vaccination would cause physicians much concern, since HZ is common in day‐to‐day patient care, and supposedly more prevalent in elderly people who should receive influenza vaccines. However, studies using a large database should be able to reveal this unexpected increase in HZ with greater certainty. As an example, it is not surprising that HZ had not been considered a monitoring target during the initial international clinical trials of the COVID‐19 vaccine.^11^, ^12^, ^13^, ^14^, ^15^ The following massive vaccination supports that there is an increased risk of HZ following COVID‐19 vaccination, which is unlikely to be coincident.

It is unclear whether influenza vaccines directly or indirectly reactivate VZV from its latent state. Immunization may impose significant psychological and physical stresses. Stress alone, however, is unlikely to be the sole cause. As discussed in the literature regarding COVID‐19 vaccines, there is speculation that dysregulation of T cells and cellular immunity,^16^ which are supposed to occur due to immune modulation caused by the target vectors^16^ or adjuvant^17^ of the vaccines, might be involved in reactivating the virus. It is also speculated that the reactivation of VZV is caused by the interaction of various factors with a summation effect. It is worthily noted that mRNA‐based vaccines are associated with HZ more frequently than other COVID‐19 vaccine types, suggesting that different types of vaccines are associated with different risks. Further studies assessing the risk of HZ following different types of influenza vaccinations may be needed.

Zoster vaccines are effective in preventing HZ and is recommended for routine use in the elderly. Both conventional HZ vaccines (Zostavax® and Shingrix®) can be safely administered concomitantly with other vaccines, such as influenza^18^, ^19^ and pneumococcal vaccines.^20^, ^21^ In Taiwan, there is no policy requiring routine use of zoster vaccine. The only zoster vaccine available in Taiwan until December 2021 was Zostavax® (a live viral vaccine from Oka/Merck, MSD), launched in December 2013 and self‐funded (without government subsidies). This vaccine is recommended for people over 50 years of age and costs about NT$ 5000 (equal to about 160–170 US dollars). The relevant information about zoster vaccine recipients cannot be obtained from the NHIRD, since the vaccine is purchased privately. Although the exact coverage rate of zoster vaccination is unknown, the Taiwan Food and Drug Administration (FDA) confirmed that 27,695 HZ vaccines were imported during mid‐2015 to mid‐2016 (assuming injection in 2016) according to the open data available on the FDA website (https://data.fda.gov.tw/frontsite/data/DataAction.do?method=doDetail&infoId=102). Since zoster vaccines were administered in smaller numbers compared to those for influenza vaccines, the effect of zoster vaccine was less likely to be a confounding factor. The clinical significance and efficacy of prior administration or simultaneous administration of influenza and HZ vaccines are worth to study in the future.

This study has several limitations. First, we only examined those who were immunized with influenza vaccine in 2016 and not an extended period or different types of influenza vaccines. The brand and manufacturer for influenza vaccines can change every year, which affects the risk from year‐to‐year. The influenza vaccines used in Taiwan since 2001 were all denatured virus‐based vaccines. Therefore, we did not extend our study period further. Second, it was difficult to pinpoint the exact onset date of their HZ from insurance database information, and patients with HZ may not seek immediate medical attention when they develop clinical symptoms. However, we confirmed the diagnosis using administration of antiviral drugs combined with the presence of diagnostic codes. According to the NHI, antiviral drugs are only reimbursed for patients who develop skin blisters within 72 h of administration. As a result, a delayed period between diagnosis and the onset of symptoms of HZ should be less than 3 days according to information from NHIRD. Even though there may be a few days of error, the risk period in this study was 2 weeks; thus, differences would not significantly affect the results. As a final point, concomitant vaccination with influenza vaccines and other types of vaccines (such as the pneumococcal vaccine) has been the trend in public health. Whether the combined use of vaccines affects the risk of zoster becomes an intriguing question. Since only a small number of subjects enrolled in our study (less than 1%) simultaneously received pneumococcal vaccine, the effect could not be assessed.

In conclusion, patients receiving influenza vaccine, denatured virus‐based, appear to have a greater marginal risk of developing HZ in the immediate 1–15 days after vaccination. We suggest that physicians should pay attention to the higher risk of developing HZ within first 2 weeks after receiving influenza vaccine.

## AUTHOR CONTRIBUTIONS

**Kai‐Che Wei:** Conceptualization; writing‐review and editing. **Yu‐Chia Chang:** Data curation; writing‐original draft preparation. **Yu‐Tung Huang:** Methodology (lead); formal analysis (lead); writing‐review and editing (supporting).

## CONFLICTS OF INTEREST

The authors declare no conflicts of interest.

### PEER REVIEW

The peer review history for this article is available at https://publons.com/publon/10.1111/irv.13055.

## Data Availability

Data cannot be shared publicly because of the policy by the Health and Welfare Data Center (HWDC), MOHW based on the Personal Data Protection Act (contact information for data application: https://dep.mohw.gov.tw/dos/cp-5119-59201-113.html). All databases were encrypted due to privacy concerns but linkable for research purposes and limited to use at the HWDC only.
